# Noise–Seeded Developmental Pattern Formation in Filamentous Cyanobacteria

**DOI:** 10.3390/life8040058

**Published:** 2018-11-09

**Authors:** Rinat Arbel-Goren, Francesca Di Patti, Duccio Fanelli, Joel Stavans

**Affiliations:** 1 Department of Physics of Complex Systems, Weizmann Institute of Science, Rehovot 7610001, Israel; rinat.goren@weizmann.ac.il; 2 Consorzio Interuniversitario Nazionale per la Scienza e Tecnologia dei Materiali, Dip. di Chimica, Università degli Studi di Firenze, Via della Lastruccia 3-13, 50019 Sesto Fiorentino, Firenze, Italy; f.dipatti@gmail.com; 3 Istituto Nazionale di Fisica Nucleare, Sezione di Firenze, via G. Sansone 1, 50019 Sesto Fiorentino, Firenze, Italy; duccio.fanelli@gmail.com; 4 Centro Interdipartimentale per lo Studio delle Dinamiche Complesse, via G. Sansone 1, 50019 Sesto Fiorentino, Firenze, Italy; 5 Dipartimento di Fisica e Astronomia, Università degli Studi di Firenze, 50019 Sesto Fiorentino, Firenze, Italy

**Keywords:** filamentous cyanobacteria, developmental pattern formation, demographic noise, stochastic Turing patterns

## Abstract

Under nitrogen-poor conditions, multicellular cyanobacteria such as *Anabaena* sp. PCC 7120 undergo a process of differentiation, forming nearly regular, developmental patterns of individual nitrogen-fixing cells, called heterocysts, interspersed between intervals of vegetative cells that carry out photosynthesis. Developmental pattern formation is mediated by morphogen species that can act as activators and inhibitors, some of which can diffuse along filaments. We survey the limitations of the classical, deterministic Turing mechanism that has been often invoked to explain pattern formation in these systems, and then, focusing on a simpler system governed by birth-death processes, we illustrate pedagogically a recently proposed paradigm that provides a much more robust description of pattern formation: stochastic Turing patterns. We emphasize the essential role that cell-to-cell differences in molecular numbers—caused by inevitable fluctuations in gene expression—play, the so called demographic noise, in seeding the formation of stochastic Turing patterns over a much larger region of parameter space, compared to their deterministic counterparts.

## 1. Introduction

A remarkable feature of developmental programs in multicellular organisms is the exquisite precision they can achieve [1,2]. Spatio-temporal gene expression pathways follow well-defined blueprints, ensuring that cells within a developmental field reach their developmental fates with high accuracy. This precision is startling in light of the noisy nature of gene expression processes [3]. Experiments carried out within the past two decades, primarily in unicellular organisms, have revealed that cells having identical genomes can display large phenotypic differences, even when exposed to the same environment [4,5,6,7,8]. Cell-to-cell variations behind these phenotypes derive in large part from the inherent stochastic nature of molecular processes at all stages of gene expression, leading to differences in mRNA and protein levels between cells, also called demographic noise. The above considerations raise the following questions: How do cells cope with these huge fluctuations and noise to make the proper decision regarding their developmental fates in multicellular organisms? How is noise filtered or alternatively, taken advantage of, in order to achieve the robustness and precision observed in developmental patterns?

Enormous progress in characterizing noise and understanding its sources has been achieved primarily in studies of unicellular micro-organisms, in which fluctuations in gene expression can be easily accessed experimentally, both at the transcriptional and post-transcriptional levels [9]. A combination of experiments and modelling has demonstrated that demographic noise is due in large part to transcriptional and translational bursting. Rather than being produced at a constant rate, as expected from a Poisson process, mRNAs are produced in transcriptional bursts as if a gene undergoes transitions between active and inactive states. Similarly, proteins are produced in bursts from individual transcripts before the latter degrade [10]. In unicellular micro-organisms, noise can play a positive role in survival under severe stresses, fluctuating environments and in the optimization of resource uptake [11,12,13]. In a developmental context, noise has proven to be essential to understand the lysis-lysogeny decision of bacteriophage lambda [14,15,16,17], as well as the initiation of competence in *B. subtilis* [18].

In contrast to unicellular micro-organisms, progress in understanding the role of noise in the formation of developmental patterns in multicellular organisms has been more limited. The idea of canalization, whereby an organism follows inexorably a “Waddington epigenetic landscape” as it forms, has been the prevailing paradigm accounting for the robustness of developmental patterns to noise and/or varying conditions [19]. While this deterministic view of development has been challenged, in no small part due to the appreciation that noise may allow cells to overcome landscape barriers and explore alternative pathways as they decide their developmental fate [20], noise has mostly been regarded as a nuisance that needs to be buffered and filtered out [3,21], e.g., by spatio-temporal averaging and spatial correlations [22]. Yet, much evidence that points to the important role that noise can play in development has accumulated. For example, recent studies have indicated that the emergence of lineage can be preceded by large expression heterogeneities [23], and a pluripotent state in embryonic stem cells is best described as an excitable system driven by transcriptional noise that generates dynamic heterogeneities at the population level [24].

A new paradigm of pattern formation, the so-called stochastic Turing patterns, based on an extension of a reaction-diffusion scheme originally envisioned by Turing to include noise, has emerged in recent years [25,26,27,28]. Turing originally showed that a deterministic, minimal model that included only two species, a rapidly diffusing inhibitor and a slowly diffusing activator, can exhibit stable, non-homogeneous spatial patterns. However, these patterns appear only in very limited regions of parameter space, and different patterns may form depending on initial conditions, for the same parameters values. These sensitivities of the classical Turing mechanism, which have been referred to as the fine-tuning problem, make the Turing model a non-robust description of pattern formation [29]. In contrast, stochastic Turing patterns can arise over regions of parameter space in which the homogeneous state is stable. Demographic noise continuously excites slowly relaxing spatial Fourier modes, exposing the lengthscale of those modes that are least stable. By forming over larger regions of parameter space, noise-seeded, stochastic Turing patterns provide a much more robust description of pattern formation than their deterministic counterparts. Stochastic Turing patterns have been shown to be relevant to systems as varied as developmental pattern formation [30,31], the dynamics of hallucinations [32], ecology [33] and biofilms [34].

**Figure 1:**
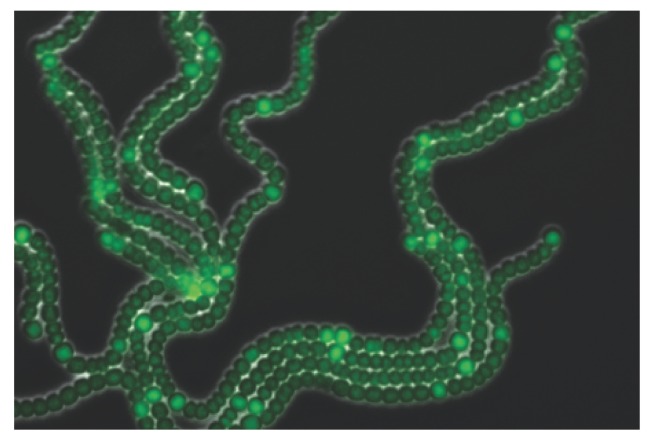
**Developmental patterns in*****Anabaena*****under nitrogen-poor conditions**. Typical snapshot of filaments expressing fluorescence from a P${}_{hetR-gfp}$ fusion, 24 h after exposure to nitrogen-poor conditions. The picture is an overlay of GFP fluorescence on a phase contrast image of the same filament. The image illustrates a pattern of individual heterocysts exhibiting high fluorescence intensity, separated by intervals of vegetative cells exhibiting low GFP fluorescence.

We have recently proposed a theoretical model to describe pattern formation in *Anabaena* sp. PCC 7120 [30], a filamentous, multicellular cyanobacterial organism that can exhibit alternative lifestyles [35,36,37]. In nitrogen-rich environments, all cells in *Anabaena* filaments carry out both oxygenic photosynthesis and fixation of combined nitrogen sources. However, when these sources become scarce, *Anabaena* can fix atmospheric nitrogen using an enzyme whose function is abolished by minute amounts of oxygen. *Anabaena* solves the incompatibility between photosynthesis and nitrogen fixation processes by the emergence of division of labor among its cells: some of them differentiate into heterocysts that specialize in nitrogen fixation but carry out no photosynthesis nor divide, whereas the rest continue to carry out photosynthesis and divide. A developmental pattern of individual heterocysts separated by nearly regular intervals of about 10–15 vegetative cells forms, with heterocysts supplying surrounding vegetative cells with fixed nitrogen products, while receiving carbohydrate products from their neighbors in return (Figure 1). This characteristic lengthscale is independent of filament length [38,39], and well-developed filaments grow by the growth and division of vegetative cells. When a vegetative cell interval becomes large enough, a new intercalary heterocyst forms in its midst, thereby maintaining the characteristic lengthscale of the developmental pattern. This organization represents one of the earliest experiments on multicellularity and differentiation on Earth, and can be traced back more than 2 billion years [37,40].

**Figure 2:**
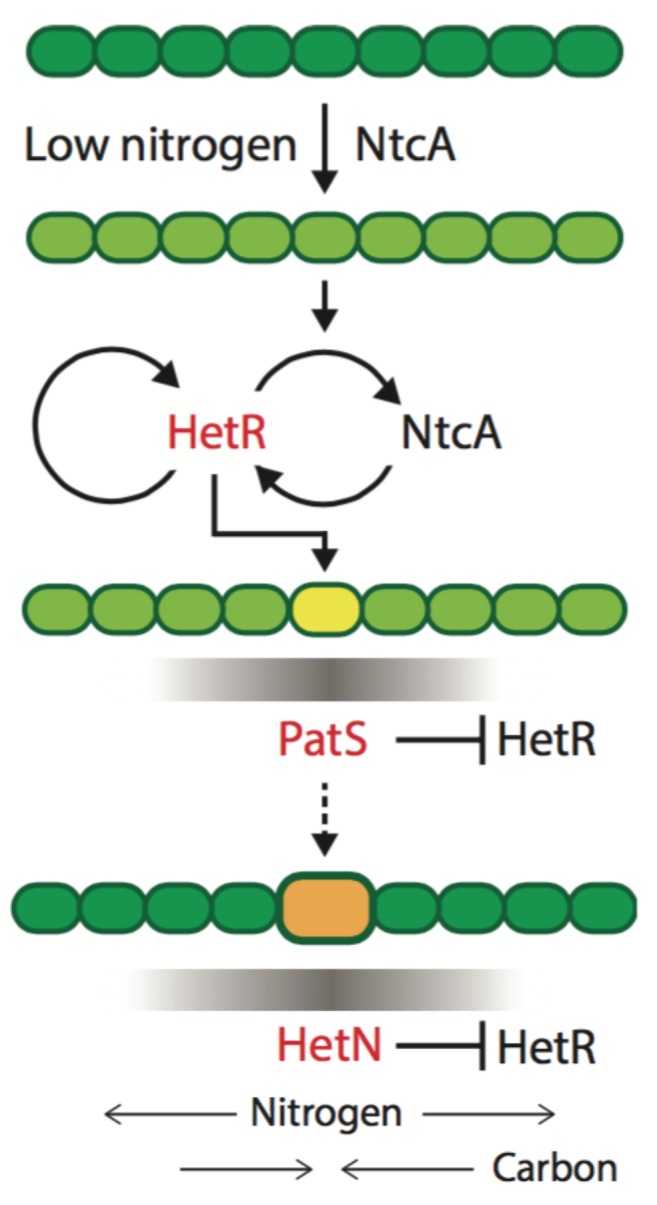
**Minimal regulatory network leading to heterocyst differentiation**. Following combined nitrogen deprivation, activation of the NtcA protein leads to the expression of HetR in some cells. NtcA and HetR undergo mutual amplification, resulting in increased levels, and in addition, HetR positively regulates its own production. During the early stages of differentiation, HetR induces expression of PatS in cells that can potentially form heterocysts (yellow). A PatS-derived peptide signal is thought to diffuse to neighboring cells (grey gradient), where it interferes with the DNA binding activity of HetR, causing its degradation and creating HetR gradients along filaments. At late stages of the differentiation process (dashed arrow), HetN is produced in heterocysts (orange), and a HetN-derived signal is conveyed to neighboring cells (grey gradient), where it inhibits HetR function and heterocyst formation. Please note that both PatS and HetN are active during pattern maintenance (29). Taken from [30]

The theoretical model of patterning in *Anabaena* we proposed is built around three dynamical variables: a non-diffusing activator protein HetR, and two proteins, PatS and HetN, whose derived products inhibit HetR function in neighboring cells, causing its degradation (Figure 2). These two HetR inhibitors have different spatio-temporal roles and are therefore not redundant [30]. Furthermore, experimental evidence was presented indicating that the numbers of HetR molecules per cell are small—a few tens—suggesting that dynamic variables cannot be treated as continuous. Lastly, gene expression in *Anabaena* is inherently noisy [41], with noise levels being comparable to those in unicellular bacteria [42]. The dynamics of a system in which variables appear in small numbers and therefore change discretely is described by master equations. Master equations embody different molecular processes such as molecule production, degradation, activation and inhibition, each one of which is characterized by specific rates. Master equations can only be solved exactly in very few cases, and therefore different schemes to obtain approximate analytical and numerical solutions have been devised. One such scheme is the celebrated van Kampen’s system size expansion [43], in which the effects of demographic noise emerge in a perturbative fashion.

The aim of the present work is to review our results in a pedagogic fashion [30], and to elaborate on the importance of stochastic contributions, as stemming from endogenous finite size corrections. To this end, we shall begin by discussing a simple birth/death reaction scheme, which will serve as a reference case to set the mathematical scene. As we will show, demographic noise results in a perturbation to the idealized deterministic evolution. Fluctuations impact indeed the dynamics by producing a seemingly unstructured modulation to the average trajectory, which can be quantified in terms of its associated statistical properties. In more complex applications, the endogenous stochasticity gets self-consistently amplified, yielding almost regular oscillations, termed quasi-cycles in the literature. Noise can hence organize in regular quasi-periodic orbits, building macroscopic order from microscopic disorder. This is a counterintuitive observation which might prove crucial for all those systems characterized by low copy numbers. We will briefly touch upon this point, by providing numerical evidence of quasi-cycles in a minimal stochastic model of intracellular calcium dynamics. In complete analogy, stochastic Turing patterns can develop when noise is significant inside spatially extended systems, such as *Anabaena*.

## 2. Macroscopic Order from Microscopic Disorder: From Birth/Death Processes to *Anabaena*’s Stochastic Patterns

In the following, we shall begin by discussing a simple stochastic model which bears pedagogical interest. The idea is to introduce the reference mathematical techniques that are routinely employed to study fluctuations in stochastic frameworks, beyond the idealized deterministic (or mean field) limit. We shall then proceed by reviewing more complex settings where the noise is shown to significantly impact the ensuing dynamics. This includes the problem of pattern formation in *Anabaena*.

**A system governed by birth/death processes.** We will consider a simple stochastic model which involves just one species. The number of individuals is dynamically modulated in time by two distinct processes, birth and death [44]. A single birth event is encoded in the following chemical equation:

(1) $$E\overset{b}{\to }X$$

where *E* is a void, i.e., an empty space, which can be eventually occupied by the newborn element. The birth event occurs with a rate *b*. Similarly, the death of an individual, which occurs at a rate *d*, can be described by:

(2) $$X\overset{d}{\to }E$$

Notice that the dying individual frees an empty void. The rules for the time evolution of the individuals within the system are formulated probabilistically. Hence, state variables are discrete and, by definition, the model is inherently stochastic. We shall here denote by *n* the number of *X* individuals and label with $n_{E}$ the number of voids *E*. The quantity $N=n+n_{E}$ is therefore constant, as voids and elements of the species transform into each other, with a one-to-one stoichiometry.

Given the inherent stochasticity of the occurrence of such events, one would like to describe the probability P(n,t) of having *n* individuals at time *t* and its evolution. To write down an equation governing this evolution, one needs the transition rate $T(n^{\prime }|n)$ from an initial configuration having *n* individuals to a final one with $n^{\prime }$. In a well-mixed situation, i.e., assuming that species are uniformly distributed, the probability of selecting an individual from *N* is n/N. Since individuals die out with a probability per unit of time *d*, the transition rate from state *n* to state n−1 reads:

(3) $$T\left(n-1|n\right)=d\frac{n}{N}$$

Analogous considerations apply to the birth reaction. The probability of selecting a void is given by $\frac{n_{E}}{N}$. Recalling the conservation law $N=n+n_{E}$ and multiplying the selected probability for the associated birth rate *b*, yields the following expression:

(4) $$T\left(n+1|n\right)=b\frac{n_{E}}{N}=b\left(1-\frac{n}{N}\right)$$

**Figure 3:**
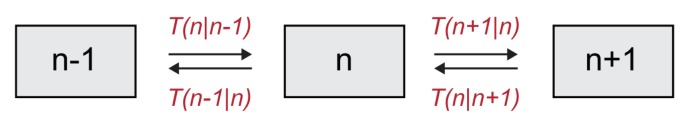
Transitions between different states in a birth-death process and associated rates. A system whose dynamics are governed by a birth-death process is characterized by the number of individuals in the system, which can change discretely. Transitions between different states (arrows) are governed by rates.

Assuming the dynamics of the system is Markovian, i.e., that there is no memory of previous events before the last one, the above transition rates allow us to write the master equation for the evolution of P(n,t). The master equation describes analytically the temporal evolution of the probability density of being in a particular state *n*, and as such, it encodes the full stochastic dynamics. For the system under consideration (Figure 3), one readily gets:

(5) $$\frac{dP(n,t)}{dt}=-\left[T(n-1|n)+T(n+1|n)\right]P\left(n,t\right)+T\left(n|n-1\right)P\left(n-1,t\right)+T\left(n|n+1\right)P\left(n+1,t\right)$$

Please note that the first term, with a negative sign in front, describes processes that decrease the value of P(n,t), (outward arrows in Figure 3), while the second and third terms, which are positive, describe increases in P(n,t) (incoming arrows in Figure 3). We remark that although we are here solely focusing on the case of a single species, the above formalism naturally extends to a setting where multiple species are to be accounted for and the system is defined on a spatially extended collection of mutually coupled domains.

**Figure 4:**
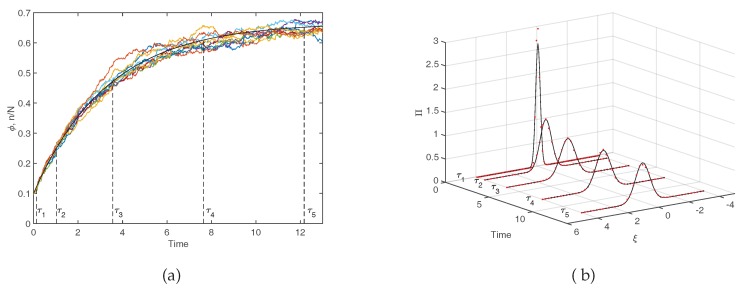
Stochastic and deterministic simulations. (**a**) Comparison between the determinsitic solution of Equation (7) (solid black line) and 10 stochastic simulations (noisy colored lines) obtained through the Gillespie algorithm [45]. The deterministic solution is explicitly given by $\phi \left(\tau \right)=\frac{b}{d+b}\left[1-\left(1-\phi_{0}\frac{b+d}{d}\right)\exp\left(-b\tau \right)\right]$. (**b**) Histograms of stochastic fluctuations ξ (red dots) obtained from 10,000 stochastic simulations. The solid black line originates from the solution of the Fokker-Planck Equation (8) which can be written as $\Pi \left(\xi ,\tau \right)=\frac{1}{\sqrt{2\pi \eta (\tau )}}e^{-\frac{\xi^{2}}{2\eta^{2}}}$ where $\eta \left(\tau \right)=\frac{bd}{{\left(b+d\right)}^{2}}-\frac{b(d-b)}{{\left(b+d\right)}^{2}}\left(1-\phi_{0}\frac{b+d}{b}\right)e^{-(b+d)\tau }-\left(\frac{b^{2}}{{\left(b+d\right)}^{2}}+\phi_{0}\frac{d-b}{d+b}\right)e^{-2(b+d)\tau }$. For both panels b=0.1, d=0.1 and $\phi_{0}=0.1$.

The master equation is ultimately a balance equation that returns the probability for observing the system in a given state, at a given time. It contains more information that usual ordinary differential equations, which are customarily invoked, as follows the law of mass action, to track the time evolution of the average population density. This latter can be recovered by taking the average of n/N (see [44] for more details): the averaging washes out the stochastic fluctuations and one is eventually left with the deterministic mean field equation for the dynamics under consideration. However, fluctuations do matter in many cases and it is hence essential to characterize them thoroughly. In the deterministic limit, the probability distribution, P(n,t) is a spike positioned at n=〈n〉, where 〈n〉 stands for the average of *n*. Starting from this observation, it can be reasonably hypothesized that the spike (a delta function, in technical jargon) deforms into a Gaussian, when fluctuations are to be accounted for. The width of the Gaussian is expected to scale as $1/\sqrt{N}$, from the central limit theorem, which amounts to effectively setting:

(6) $$\frac{n}{N}=\phi +\frac{\xi }{\sqrt{N}}$$

where ϕ=〈n〉/N and ξ is a stochastic variable. The former ansatz defines the so-called van Kampen system size expansion: the quantity $1/\sqrt{N}$ is small for moderately large (but not infinite) systems and it can be used as a perturbative parameter. Performing the van Kampen expansion is technically involving and the mathematical details are here deliberately omitted. The interested reader can however refer to [44] for further information on the implementation of the method, with reference to the case at hand. At the leading order of the expansion one eventually recovers the deterministic mean field equation, namely the ordinary differential equation that governs the time evolution of the average density ϕ:

(7) $$\frac{d\phi }{d\tau }=b-\left(b+d\right)\phi$$

where τ=t/N is the rescaled (macroscopic) time. Please note that the second term includes *b* because we are considering the empty spaces, whose concentration is 1−ϕ (see Equation (4). The solution of this equation, given explicitly in the caption of Figure 4 converges exponentially to the steady–state value $\phi^{*}=b/\left(b+d\right)$. Equation (7) reflects in an increasingly accurate fashion the behavior of the system as *N* tends to very large values. Equivalently, it matches the observation when averages are performed over a large sample of independent realizations of the stochastic dynamics. This fact is exemplified in the left panel of Figure 4 where the deterministic solution, as stemming from Equation (7), is compared to a limited set of stochastic trajectories. The inherent stochasticity, arising from finite size corrections, blurs the deterministic outcome, and materializes in erratic fluctuations around the predicted mean field concentration. The main effect of demographic noise can be statistically captured by pushing the van Kampen expansion to the next to leading order. The fluctuations can be shown to obey a linear stochastic equation of the Langevin type, which is parametrized in terms of the chemical rates that enter the definition of the microscopic reactions (1) and (2). Formally, the Langevin equation is equivalent to the following Fokker-Planck equation for the probability distribution Π(ξ,t) of the fluctuations ξ:

(8) $$\frac{\partial }{\partial \tau }\Pi =\left(d+b\right)\frac{\partial }{\partial \xi }\left(\xi \Pi \right)+\frac{1}{2}\left[d\phi +b\left(1-\phi \right)\right]\frac{\partial^{2}}{\partial \xi^{2}}\Pi$$

In other words, the stochastic perturbations around the averaged (deterministic) dynamics are statistically distributed according to Π(ξ,t), a function that is modulated over time and which can be directly accessed by solving the above Equation (8). As anticipated (see caption of Figure 4), the distribution of fluctuations is predicted to be a Gaussian. The width of the Gaussian changes, as time progresses. In Figure 4b the theoretical Π(ξ,t) is calculated numerically, by sampling the stochastic dynamics at different times. More specifically, we run a large set of stochastic simulations, freezing the reactive parameters to the nominal values as specified in the caption, and computing the frequency of occurrence of the fluctuations, with an imposed binning, at the selected time of measurement. Theory and simulations are in excellent agreement, as testified by visual inspection of Figure 4b. In conclusion, the van Kampen system size expansion provides a viable tool to reliably assess the role played by demographic fluctuations in the framework of a stochastic model, beyond the simplistic deterministic viewpoint that is customarily adopted.

In the pedagogical example that we have here addressed, however, fluctuations do not deeply affect the deterministic dynamics. They simply materialize as a local perturbation to the mean field dynamics, without altering the underlying scheme. In more complex cases, i.e., when more than one species are involved, fluctuations can yield non trivial and unexpected outcomes, as we shall comment in the following. Interestingly, the method due to van Kampen, and illustrated above with reference to a simple birth and death reaction scheme, can be readily adapted to account for these generalized scenarios.

**Figure 5:**
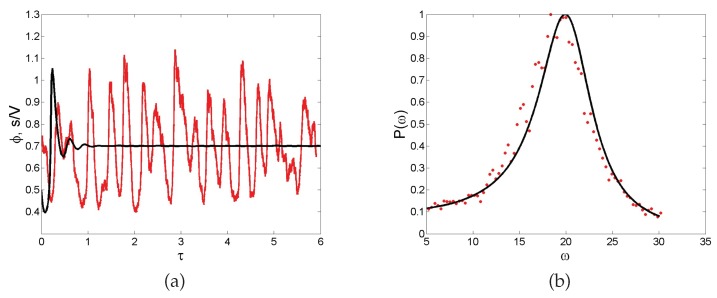
(**a**) Time evolution of the intracellular calcium concentration ϕ. The black solid line which converges to an asymptotic stable fixed point refers to the integration of the deterministic system while the wiggling curve (red online) follows stochastic simulations. (**b**) The power spectrum of fluctuations as a function of the frequency of the temporals oscillations ω. The solid line stands for the theoretical prediction while the symbols refer to the stochastic simulations averaged over 200 independent realizations. Both figures are from [47].

**Noise-sustained oscillations.** A common feature of individual-based models with two or more degrees of freedom is for instance to excite macroscopic scale coherent oscillations, termed in the literature quasi-cycles. Consider a stochastic model that is bound to converge deterministically to a trivial fixed point (constant equilibrium solution) via damped oscillations. Then, the endogenous (demographic) noise is amplified by a resonance mechanism, resulting in large amplitude oscillations that are not displayed by the deterministic dynamics. Stated differently, the inherent stochasticity results in a robust mechanism that sustains cyclic behavior, also when the deterministic conditions for the onset of periodic solution are not met. As an example, we display in Figure 5a the time evolution of the concentration of intracellular calcium, as obtained from a stochastic version of a celebrated deterministic model due to Goldbeter [46]. Without entering into the details of the model, we can immediately appreciate from visual inspection of Figure 5a the persistent oscillations executed by the stochastic trajectory. Conversely, the deterministic solution (black solid line) approaches the asymptotic state, once the initial oscillatory transient fades away. Rather than being characterized by a single period, the stochastic cycles oscillate with a distribution of periods centered on an average value. This can be seen by performing a Fourier transform in time of the recorded stochastic signal: by taking the modulus squared of the Fourier transform, one obtains a smooth function spread about the characteristic frequency of the system. This is the so-called power spectrum, a powerful diagnostic tool which is plotted, for the application here reviewed, in Figure 5b. Symbols stand for the power spectrum computed after direct simulations, while the solid line refers to the analytical calculation (not detailed here) which follows again the van Kampen recipe. The spread of the power spectrum, which can be accessed analytically, defines the degree of coherence of the displayed oscillations. The narrower the power spectrum, the more regular the oscillations. Macroscopic oscillations, seemingly regular in time, can therefore originate from a noisy microscopic perturbation, which stems from the intimate discreteness of the system being explored.

**Figure 6:**
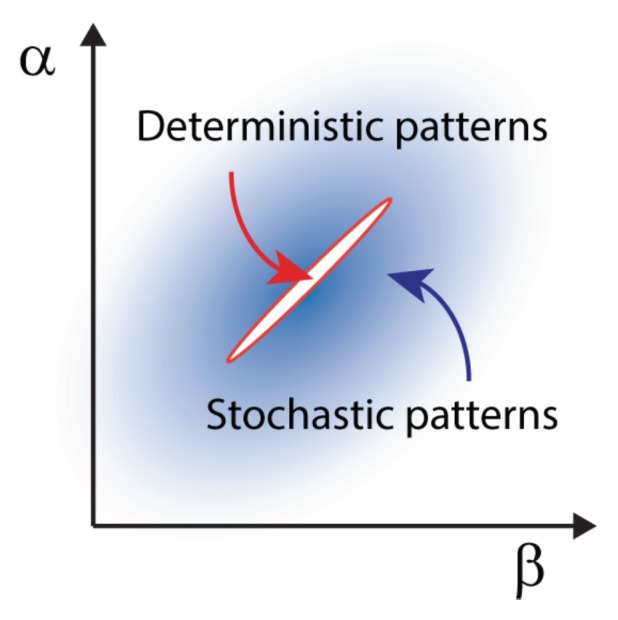
Schematic parameter space where deterministic and stochastic Turing patterns appear. Deterministic Turing patterns typically appear only within a small region of parameter space (α,β), bounded in orange. Here α and β are two parameters controlling rates in the dynamical equations. Outside this region, stochastic Turing patterns appear with different likelihood, here coded by different shades of blue.

**Stochastic Turing patterns and*****Anabaena***. This intriguing concept, illustrated in the previous section for the temporal domain, can be extended to the realm of spatial systems. In this case, distinct constituents interact and relocate in space following the rule of diffusion. Demographic noise can seed the emergence of sustained oscillations in space, veritable patterns characterized by a dynamically selected wavelength that can be quantified under the van Kampen approach. Stochastic Turing patterns, as they are refereed to, are more robust than their deterministic analogs and extend over a larger portion of the reference parameter space, as schematically outlined in Figure 6. From an operational perspective, predicting the existence of the stochastic Turing patterns amounts to computing the power spectrum of fluctuations at the next to leading order in the van Kampen expansion. When the system is spatially extended, the power spectrum depends on two sets of frequencies, respectively associated with space and time. A peak in the power spectrum against the spatial wavenumber *k*, signals the presence of a noise-driven pattern.

**Figure 7:**
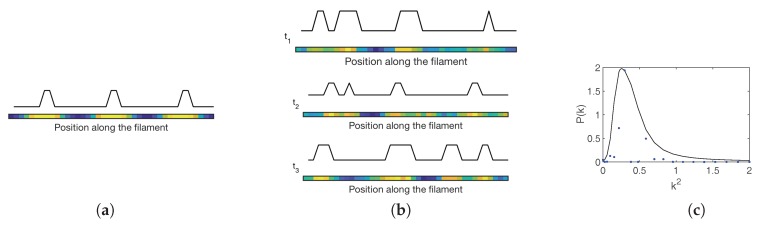
Deterministic and stochastic Turing patterns in an *Anabaena* model. (**a**) Steady-state concentration of HetR as a function of position along a filament, as determined from a solution to the model within the deterministic region. Yellow (blue) denotes regions where HetR concentration is high (low). The solid black line represents possible future locations where commitment will take place and an heterocyst will be formed. Please note that HetR is high in a region that may comprise several cells, as no commitment is enforced in the model. (**b**) Snapshots of stochastic patterns at three different times. (**c**) Power spectrum relative to stochastic Turing patterns as a function of the wavenumber *k*. The solid black line corresponds to the theoretical prediction on a continuous domain, and blue stars stand for the power spectrum obtained from one realization of the Gillespie algorithm (snapshot at time $t_{3}$ displayed in panel (b)).

Owing to their flexibility, stochastic Turing patterns possibly define a universal paradigm for the spontaneous generation of spatial ordering in reaction-diffusion systems. The model of pattern formation in *Anabaena* discussed in [30], elaborates along these lines. A set of chemical equations are introduced that encode the interaction among individual HetR, PatS and HetN signals. The diffusion of mobile species across the one-dimensional filament is also modeled via chemical equations. The stochastic dynamics of the system is hence traced back to a master equation, which can be analyzed with the van Kampen machinery. At the leading order of the expansion one recovers the ordinary differential equations for the evolution of non-fluctuating variables, i.e., the spatial concentration of the three species involved. Deterministic Turing patterns can in principle develop (see Figure 7a) in a restricted region of the parameter space, which is too small to account for patterns that are observed in nature. In line with the above, carrying out the analysis at the next-to-leading order in the van Kampen approximation enables one to expose the existence of stochastic Turing patterns, outside the region of the parameter space where the deterministic patterns form. Interestingly enough, stochastic patterns also set in when the diffusion constants associated with mobile species are very close, suggesting a novel robust scenario for the formation and maintenance of developmental patterns in *Anabaena*. Snapshots of stochastic patterns of HetR at different times as obtained for the model of *Anabaena* using the Gillespie algorithm [45,48], are depicted in Figure 7b. The unavoidable stochasticity yields a distribution of spacings around the value corresponding to the peak displayed in Figure 7c where the power spectrum for HetR is shown. This nearly regular spacing is characterized by a typical separation between regions of high HetR concentration that is compatible with the characteristic lengthscale of *Anabaena* developmental patterns, as seen in real experiments. The goal of the model in Ref. [30] was to demonstrate the relevance of stochastic Turing patterns as a more robust description of pattern formation in *Anabaena*, rather than to make quantitative comparisons with experiments, given that most chemical kinetic parameters in this system are unknown. It is important to note that the minimal model in Ref. [30] includes only PatS, HetR and HetN, but does not include any commitment to irreversible differentiation. Therefore regions of high HetR concentrations as those observed in Figure 7a,b are not resolved into single heterocysts. Hence, no observables such as vegetative interval size distributions can be obtained from the model and compared with experiment. It was proposed that when fluctuations in HetR concentration in specific cells reach sufficiently high levels, irreversible commitment to the formation of heterocysts may be triggered by downstream genetic processes. These latter could then enforce the stabilization of the transient stochastic Turing patterns.

## 3. Conclusions

In contrast to the view that noise plays negative roles during the development of multicellular organisms, and must be therefore buffered or filtered out (refs below), the paradigm of stochastic Turing patterns reviewed here in the case of pattern formation in *Anabaena* supports an alternative view according to which demographic noise can play an advantageous and essential role. Noise can excite long-lived spatial or temporal modes that can be resonantly amplified, seeding the formation of stochastic patterns over much larger regions of parameter space, without the need for parameter fine tuning than their deterministic counterparts. This not only creates a much richer dynamical behavior both in space and time, but also endows the system under consideration with a much more robust description.
